# Facility-based simulation as a programmatic tool for implementing a statewide contraceptive initiative

**DOI:** 10.1186/s12913-022-08332-4

**Published:** 2022-07-29

**Authors:** Susanna R. Cohen, Jami Baayd, Gabriela García, Caitlin Quade, Alexandra Gero, Madison Ekey, Catherine Poggio, Rebecca Simmons

**Affiliations:** 1 LIFT Simulation Design Lab, Division of Family Planning, Department of Obstetrics and Gynecology, University of Utah, 30 North 1900 East, Salt Lake City, UT 84132 USA; 2 Division of Family Planning, Department of Obstetrics and Gynecology, University of Utah, 30 North 1900 East, Salt Lake City, UT 84132 USA

**Keywords:** Family Planning, Simulation, Implementation fidelity, Clinic audits

## Abstract

**Background:**

Assessing implementation fidelity is highly recommended, but successful approaches can be challenging. Family Planning Elevated (FPE) is a statewide contraceptive initiative which partnered with 28 health clinics across Utah. To assess implementation fidelity, we developed in-situ high-fidelity simulation training to both determine clinic adherence to FPE and offer education to implementing teams. This study aimed to develop, pilot, and assess the use of simulation as a tool for measuring implementation fidelity.

**Methods:**

We developed two simulation scenarios to determine implementation fidelity: one scenario wherein a client is seeking a new method of contraception and another in which the same client has returned to discontinue the method. Both simulations contained multiple aspects of program implementation (e.g., determining program eligibility). We then offered simulations to all FPE partner organizations. To assess simulation training as a tool for determining implementation fidelity, we developed strategies aligned with each aspect of an adapted RE-AIM framework, including pre-post surveys, acceptability and self-efficacy testing, a checklist for programmatic adherence, field notes, action planning and analysis of monitoring data.

**Results:**

Fifteen clinical sites and 71 team members participated in the in-situ simulations. Assessment of the checklist showed that 90% of the clinics successfully demonstrated key program components, including person-centered counseling techniques such as sitting at the patient’s level (95.8%); asking open-ended questions (100%); and explaining how to use the contraceptive method selected (91.7%). More than half of clinics fell short in programmatic areas including: confirmation that the FPE program covered same-day intrauterine device insertion (54.2%), and education on health risks associated with the selected contraceptive method (58.3%). After simulation, participants reported improved knowledge of how FPE works (*p* =  < 0.001), increased ability to identify FPE-eligible clients (*p* = 0.02) and heightened self-efficacy in helping clients select a method (*p* = 0.03). Participants were satisfied with the simulations, with most (84.1%) reporting that the simulation exceeded their expectations.

**Conclusions:**

Highly-realistic in-situ family planning simulations are acceptable to participants, positively change knowledge and clinical team confidence, and can identify systems gaps in clinical care and program implementation. Simulation offers a reciprocal way of monitoring implementation fidelity of a family planning access initiative.

**Trial registration:**

This project was determined to be exempt by the IRB of the University of Utah, the larger Family Planning Elevated program under which this pilot study was nested is registered at ClinicalTrials.gov Identifier: NCT03877757.

**Supplementary Information:**

The online version contains supplementary material available at 10.1186/s12913-022-08332-4.

## Contributions to the literature:

- In-clinic simulations on family planning counseling and provision can be used with interprofessional teams in a variety of clinical settings to improve provider knowledge, self-efficacy and systems challenges.
- Simulation trainings represent a potential research and programmatic tool that can be used in the ongoing challenge to understanding how a program is being implemented in real world settings (implementation fidelity).

## Background

Unlike the tightly controlled parameters of clinical trials, public health interventions are introduced into systems that are unpredictable, dynamic, and highly complex [1, 2]. Public health interventions often operate through partnerships between those who designed and oversee the program and the sites who implement those programs. To understand if the intervention is operating as intended, the program designers need to know *how* the program is being implemented at each of the unique sites [3, 4]. Specifically, the program designers need to evaluate *implementation fidelity* (IF), or how closely the implementing sites deliver the program as intended [5]. While implementation researchers agree on the importance of evaluating IF, there are not clear best practices on how to do so [6, 7]. The most common ways of measuring IF are via self-report on fidelity measures from implementing sites, and observation of sites (via recorded sessions, or through in-person observations) [6, 8]. Both of these approaches have considerable limitations: self-reports may be biased by implementers wanting to please program designers, and are also limited by how well the implementers understand the components of the program. Observations of program delivery can also be problematic: they can influence the way participants deliver the intervention (the Hawthorne effect) [9], are logistically difficult (difficult to ensure the behaviors you want to witness will happen during your observation period), and in patient care settings may lead to concerns about patient comfort and privacy [10]. Quality audits are another tool that can be used to assess IF and improve program quality. These audits face similar logistical barriers as direct observation, and have been shown to be less effective when conducted by external entities (program designers) as opposed to those conducted by colleagues at the implementation site [10–12]. In addition, these assessments are often unidirectional, and do not allow for an equal exchange of ideas or an avenue for implementers to act in true partnership by collaborating on program design and improvement.

In 2019, the Family Planning Division at the University of Utah began implementation of a statewide contraceptive initiative, Family Planning Elevated (FPE). The Family Planning Elevated Contraceptive Access Program (FPE CAP) is the clinic-focused element of the initiative, aimed at improving contraceptive access through the health system. Clinics who were interested in participating in FPE CAP completed a detailed application. The application collected information regarding the populations served, organizational readiness to provide contraceptive care, data systems used, and each organization’s specific training and resource needs. For organizations who were accepted into FPE CAP, the program offered a tailored package for each participating organization, based on their size, populations served, budgetary needs, and training requirements. The FPE CAP package for each site included reimbursement for contraceptive methods and services for clients who fell in the coverage gap, technical assistance on contraceptive and logistical topics, education and clinical training on contraceptive provision, and cash grants for equipment and personnel expenses [13]. The program was designed with evaluative elements to ensure staff and funders could track progress that resulted from the intervention, including a year-one audit of each participating clinic to assess implementation fidelity, and correct if implementation was off course.

When determining an approach to examine implementation fidelity at each of the clinical partners sites, FPE program staff wanted to simultaneously assess fidelity *and* provide an educational benefit to the participating clinics. We wanted to measure fidelity in a way that was supportive of our relationships with the clinical sites, did not make the clinic staff feel like we were evaluating them individually, and improved sites’ ability to provide person-centered contraceptive care. Additionally, we wanted to observe specific components of programmatic fidelity in action, and we knew that simply observing routine patient visits would leave to chance which elements of the program we could observe. To meet our goals of 1) assessing implementation fidelity and 2) providing beneficial learning opportunities to the clinics, our team piloted the use of highly-realistic in-situ simulation trainings with our partner family planning clinics. Simulations with facilitated debriefs have been widely utilized to foster interprofessional teamwork and communication [14] improve operational readiness [15, 16], and increase providers’ knowledge and skills [17–19]. To our knowledge, simulation has not previously been used as a tool for measuring implementation fidelity.

## Methods

The aim of this study was to develop, pilot, and assess the use of simulation as a tool for measuring implementation fidelity. Additionally, we evaluated if the simulation trainings could benefit not only FPE’s evaluation of programmatic implementation, but also simultaneously aid the clinics in achieving program adherence, improve clinic team knowledge of programmatic elements, and provide real time feedback to program designers to inform iterative implementation. Each facility received one 4-h simulation training workshop, and participants completed pre- and post- test. Simulation trainings were conducted onsite at all primary care clinic organizations participating in Family Planning Elevated. These sites included community health centers, private clinics, and county health departments.

### Intervention overview

Family Planning Elevated and the LIFT Simulation Design Lab collaborated to create a clinic-based simulation training. Each FPE CAP organization received a simulation training after they had been participating in the program for one year (of a two-year program). Each training lasted four hours and involved multiple members of the clinical team, including clinical coordinators or managers, front-desk staff, medical assistants, and providers. The trainings included: 1) a 45-min discussion (a component of FPE’s larger process evaluation [20]); 2) two simulation scenarios aimed at understanding a clinic’s provision of key aspects of the FPE program; 3) facilitated debriefs after each scenario; and 4) an action planning session.

The process evaluation component of the simulation training involved a facilitated discussion about the current implementation of FPE at the clinic site. The process evaluator asked open-ended questions about barriers and facilitators of FPE implementation, including the aspects of FPE that have been the most difficult to implement, and programmatic successes in the first year of implementation.

**Fig. 1:**
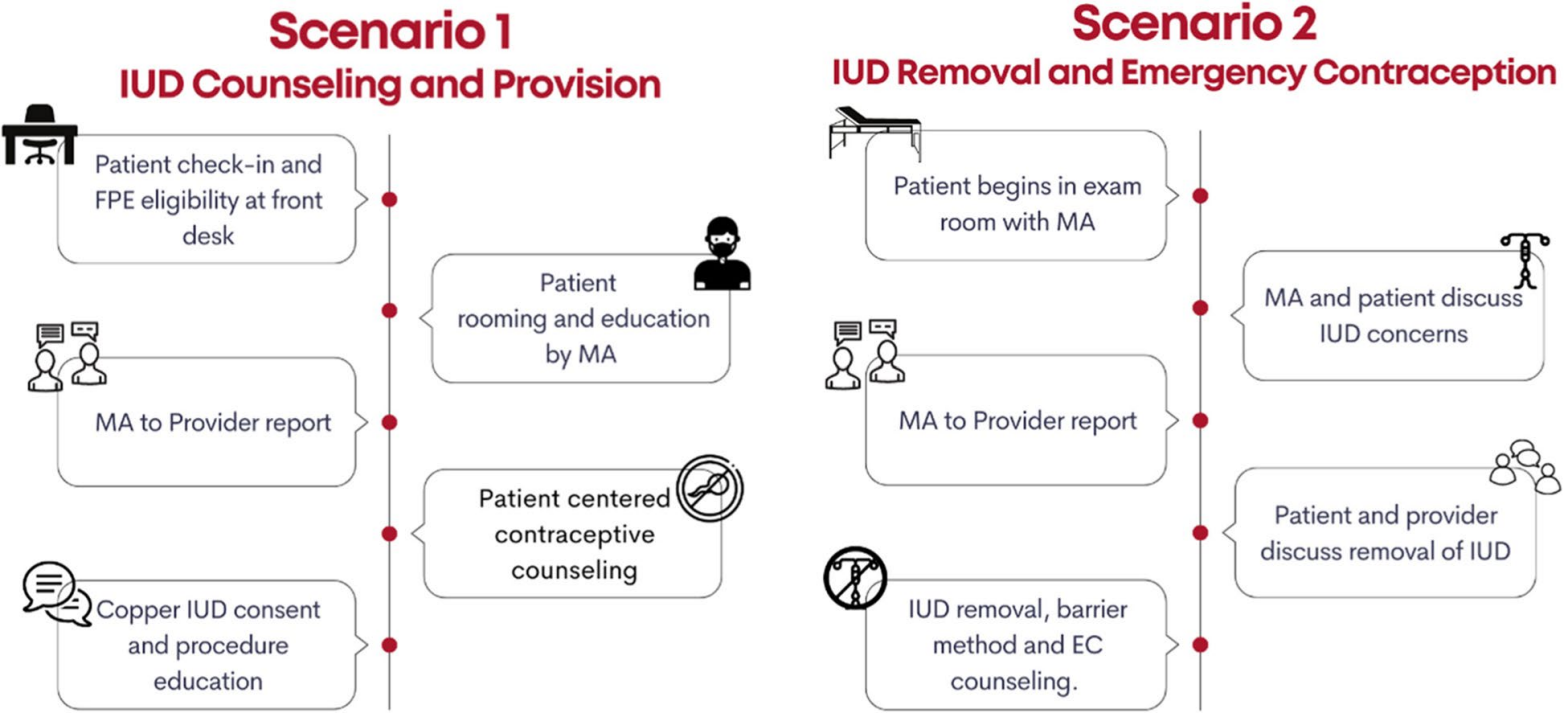
Overview of Simulation Scenarios

After the process evaluation, participants were invited to engage in the simulation scenarios. In both scenarios, we used a simulated patient actor (SP) to allow providers the opportunity to practice the clinical and interpersonal skills needed during intrauterine device (IUD) contraceptive counseling and removal appointments. See Fig. 1: Overview of Simulation Scenarios for an overview of each scenario and Table 1 for a description of how the scenarios tie to each of FPE’s learning objectives.

**Table 1:** Simulation Scenarios and Objectives

**Scenario 1:** IUD Counseling and Provision	**Patient summary:** *New patient visit for contraceptive counseling who is FPE-program eligible. Patient is nervous upon arrival to clinic. Patient ultimately selects a copper IUD*	
**Cognitive Objectives**	**Technical Objectives**	**Behavioral Objectives**
1. Know FPE eligibility criteria including classifying undocumented clients and mixed-status relationships 2. Understand the principles of person-centered counseling 3. Identify the range of contraceptive methods available 4. Understand the different types of IUDs and their mechanisms of action and side effects 5. Identify when same-day appointments are advisable	6. Classify client as FPE eligible 7. Demonstrate person-centered counseling 8. Elicit client’s contraceptive priorities 9. Demonstrate IUD pre-insertion counseling 10. Utilize the FPE eligibility job aid	11. Effectively communicate patient history to other members of the team 12. Establish rapport with client 13. Maintain patient confidentiality
**Scenario 2:** IUD Removal and Emergency Contraception	**Patient Summary:** *Return visit, patient desires IUD removal, and emergency contraceptive RX given. Patient strongly desires shift to barrier method*	
**Cognitive Objectives**	**Technical Objectives**	**Behavioral Objectives**
1. Know FPE coverage of methods 2. Understand the principles of person-centered counseling 3. Identify the range of contraceptive methods available 4. Understand the different types of IUDs and their mechanisms of action and SEs 5. Identify when same-day appointments are advisable 6. Understand the use of EC, types of EC and mechanism of action of each	7. Demonstrate person-centered counseling 8. Elicit client’s contraceptive priorities 9. Demonstrate respectful IUD removal 10. Demonstrate EC counseling	11. Open communication between provider and client 12. Establish rapport with client 13. Maintain patient confidentiality 14. Communication free of provider bias regarding method choice

**Fig. 2:**
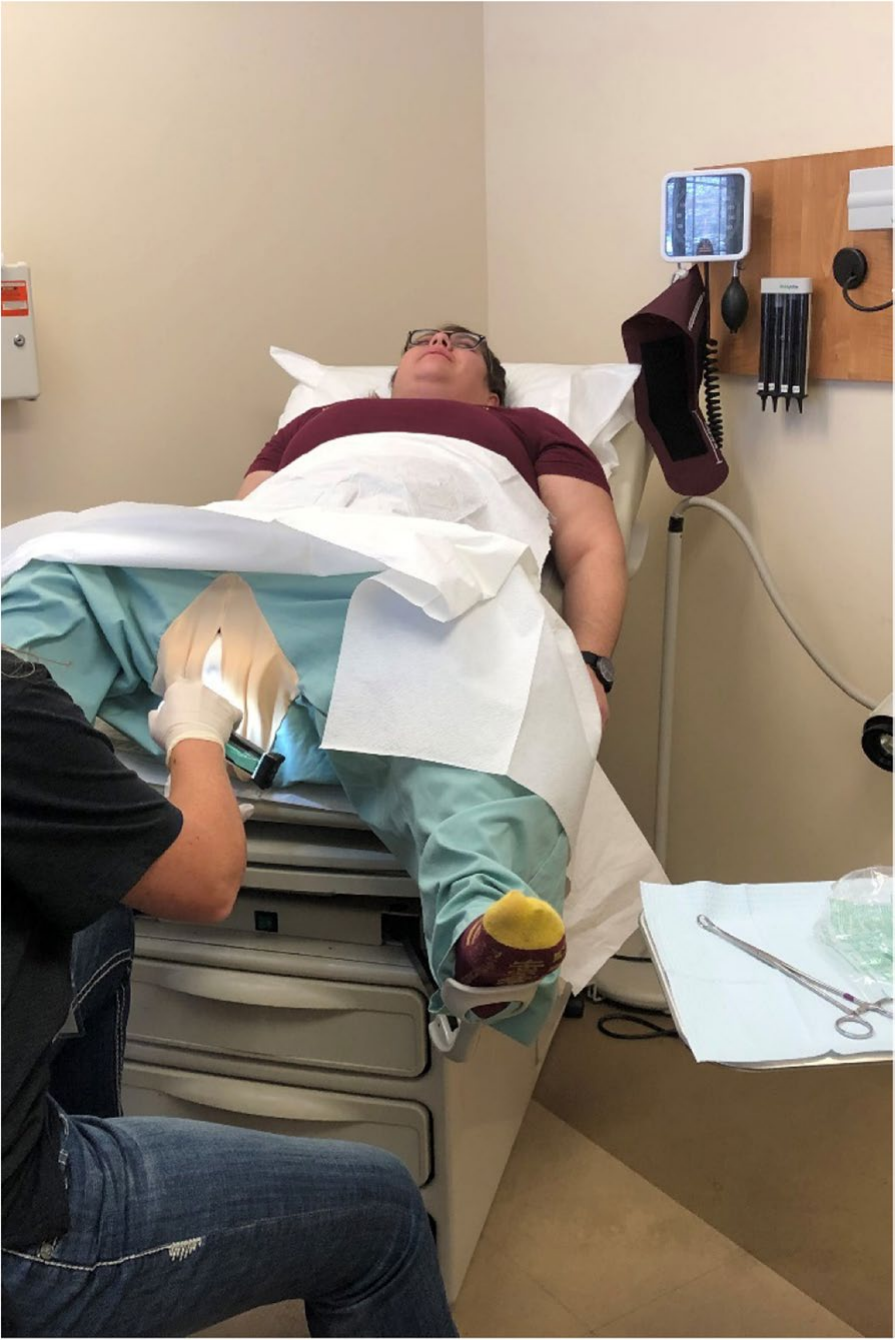
Simulated patient with PartoPants^TM^ and IUD Task Trainer

To support simulation of the IUD removal, we used a hybrid simulator called PartoPants™ [21] with an IUD task trainer inside the pants. This setup allowed the providers to demonstrate removal of the IUD from the task trainer, while coaching the SP through the process (See Fig. 2 (note picture taken in scenario testing prior to COVID-19)). The simulations were live-streamed to a conference room within the clinic so that other clinic team members could watch the scenario without having to crowd into the patient exam room. Prior to the simulation, a test patient electronic health record (EHR) was sent to clinic administration. This allowed simulation participants to use the EHR as they would during a typical patient encounter.

Both scenarios involved a patient actor (GG). During the simulation, the actor followed prompts that introduced learning objectives and known barriers to family planning service access. These prompts included asking about FPE eligibility (income and immigration status), contraceptive method efficacy, mechanism of action, method cost, and availability of same day IUD placement and follow-up. Simulations were conducted in English or Spanish (with clinic interpreters) depending on the desire of the clinic and the language typically spoken by their patients.

After each simulation, the entire clinic team participated in a facilitated debrief, discussing the scenario and talking through various aspects of the participants’ experience. This debrief provided opportunities for the FPE program team to clarify programmatic elements about FPE, educate the clinical staff on new/updated clinical best practices, and collaboratively discuss barriers to FPE implementation at the clinic site. Following the two simulations and debriefs, the clinic and programmatic teams engaged in an action planning session led by a *neutral* party (LIFT Simulation Design Lab), where both teams identified goals for improving program implementation at the site.

### Data collection

The components of this study are presented here in accordance with an adapted RE-AIM framework [22]. The RE-AIM framework was developed to understand key components of intervention implementation that may not be adequately captured through traditional assessments. Here, we use the framework to anchor our evaluation of simulation as 1) a tool for measuring implementation fidelity and 2) a useful learning opportunity for clinic sites. An overview of the study questions and how they were assessed in accordance with the framework is provided in Table 2. The study was approved by the University of Utah Institutional Review Board (IRB 00,135,600).

**Table 2:** Integration of Family Planning Elevated (FPE) simulation trainings with the RE-AIM implementation framework

Dimension and Project Specific Definition	Question Answered	Assessed through
***Reach*** Number, percentage, and representativeness of those who participated in the simulation trainings	• *Did the simulation trainings reach those who implement the FPE program at the different clinics?*	• Attendance logs • Participant demographics • Pre/Post-simulation surveys
***Effectiveness*** Intervention effects on targeted outcomes	• *Did the simulation training identify the degree of clinical compliance to key components of FPE?* • *Did the simulation trainings change participant knowledge/self-efficacy?*	• Simulation video checklist coding • Pre/Post-simulation surveys
***Adoption*** Acceptability of the intervention to the target population	• *Did clinic participants feel the simulation trainings were a good use of their time?*	• Post-simulation surveys
***Implementation*** The extent to which the simulation was consistently implemented across sites	• *Were the simulation trainings changed/adapted over time?*	• Field notes
***Maintenance*** The extent to which the simulation training impacted programmatic and clinic organizational implementation	• *Did the clinics implement FPE differently following the intervention?* • *Did FPE change programmatically based on information obtained in the simulations*	• Action planning • Monitoring data

To assess whether simulation had effects on targeted outcomes we developed a checklist of FPE-specific elements inherent in a standard contraceptive counseling visit (See Additional File 1). Checklists for each interaction component of the simulation (at the front-desk, with the MA, and with the provider) were developed, piloted, and refined by a committee of content experts. All individuals who participated in the simulation sessions consented to be video recorded as part of this assessment. Upon consent, study staff video recorded the simulations and simulation recordings were subsequently stored in a secure cloud-based folder for later analysis.

Two members of the study team who did not participate in the simulation development or implementation of the simulations (CP, ME) independently reviewed the recorded simulations and completed the simulation checklists. Prior to coding, members of the research team demonstrated the use of the checklist, oriented the coders and practiced coding. All simulations were double coded to assess for agreement between reviewers on programmatic constructs. We calculated kappa statistics to assess inter-rater agreement between coders.

### Data analysis

To evaluate the effectiveness of simulation as a learning opportunity for clinic staff, we assessed changes to knowledge and self-efficacy through pre- and post-simulation training evaluations (see Additional File 2). These assessments included questions about participant confidence in their ability to implement the FPE program, their attitudes toward contraceptive service provision, and the importance they place on providing high-quality contraceptive care as part of their clinical practice. To determine change, we conducted McNemar’s tests on each item.

Assessments of programmatic adoption utilized summary statistics of compiled post-simulation evaluation scores. Implementation changes to the simulation were captured through our formal process evaluation, [20] which uses the CFIR framework [23].

To determine whether simulation resulted in changed clinic behaviors as part of the maintenance phase, we utilized our programmatic monitoring data. This data includes the total number of FPE clients served by each clinic per month. We used paired t-tests to compare the numbers of FPE clients served, as well as the proportions of FPE clients to total contraceptive clients, for three months prior to the simulation training and three months following in order to determine whether the simulations resulted in more individuals entering the FPE program. We theorized that the simulations may increase program adherence, better prioritization of Family Planning care and greater provider/staff motivation to bring up Family Planning services. We also utilized field notes from our formal process evaluation to determine which items from the simulation action planning sessions resulted in FPE programmatic change.

## Results

### Reach

We conducted simulation trainings with each of FPE CAP’s eight organizations. While some FPE CAP organizations only had one clinic, several of the organizations had multiple clinics. When we rolled out the simulation training we required each organization to participate in a simulation at a least one clinical site, as well as extedned the offer to conduct simulations at additional clinical sites if organizations were interested. In total we conducted simulations at 15 clinical sites with a total of 71 participants across all sites. Each simulation training involved at least one care provider (physician, nurse practitioner/nurse midwife or physician assistant), one medical assistant (MA) or registered nurse, and a front desk staff member. Most trainings also included members of the clinic’s administrative team and additional providers and clinic support staff. Due to precautions around COVID-19, we limited most simulations to six or fewer clinic participants. The clinics were spread across the state with two-thirds of clinics located in an urban center vs rural community. Eight were FQHCs, 3 public health department contraceptive clinics, and 4 private practice. Forty percent of the clinics that specialize in reproductive health services, while the remaining 60% provided comprehensive health services.

### Effectiveness

Checklists were coded for a total of 15 simulation videos. To adapt to the COVID-19 pandemic, several clinics implemented programmatic changes in their approach to family planning visits, which resulted in widespread discrepancies in front-desk/MA practices across clinic sites. For example, to limit clinic staff and patient exposure, some clinics had MAs conduct the initial client intake by phone prior to the clinic visit. Because of this, we opted to limit our assessment of the checklist (and inter-rater agreement) to the provider component of the first scenario since it captured the most learning objectives and remained largely consistent despite fluctuating pandemic restrictions throughout the study period. Kappa scores for this scenario were 0.71 (0.64, 0.77), indicating substantial agreement between the reviewers.

Across the 15 simulations, coders agreed that more than 90% of the clinics were: introducing themselves to the patient (100%); sitting at the patient’s level (95.8%), asking open-ended questions (100%); asking about previous contraceptive experiences (91.7%); answering the patient’s questions in an affirming, normalizing manner (100%); clearly explaining how to use the contraceptive method selected (91.7%); and sharing clinically accurate information (100%). Coders noted that more than half of clinics fell short in a number of areas including: medical assistant did not confirm the patient could receive their selected contraceptive method same-day (54.2%), and providers did not explain the health risks associated with the contraceptive method selected by the patient (58.3%). Coders also identified programmatic areas that ultimately were coded as “not applicable” or not occurring in the simulation scenarios within clinic sites, namely: the front desk person introducing themselves (50%), the front-desk person identifying the patient’s income level as Medicaid-eligible (66.7%), the front-desk person telling the patient that they qualify for FPE (54.2%), and the medical assistant providing accurate information about the FPE program to the patient (45.8%).

To measure our second component of intervention effectiveness—participant programmatic knowledge and self-efficacy—we conducted surveys before and after each training. These.

assessments showed changes in self-reported understanding of the FPE program and beliefs in individual and clinic abilities to support contraceptive clients; for more than half of the surveys questions asked, the difference between participants’ pre/post responses was statistically significant at the 0.05 level. Table 3 provides an overview of pre/post survey findings.

**Table 3:** Participant program knowledge and self-efficacy pre- and post-survey outcomes

Statement	Pre-Simulation	Post-Simulation	*P*-value
*I have a good understanding of how contraceptive case is provided at my clinic (including how it occurs when I am not around)*			**0.05**
Strongly agree	32 (57.1%)	43 (75.4%)	
Agree	18 (32.1%)	13 (22.8%)	
Neutral	6 (10.7%)	1 (1.8%)	
Disagree	0	0	
Strongly disagree	0	0	
*I have a good understanding of how FPE works within my clinic*			** < 0.001**
Strongly agree	27 (47.4%)	46 (80.7%)	
Agree	20 (35.1%)	11 (19.3%)	
Neutral	7 (12.3%)	0	
Disagree	3 (5.3%)	0	
Strongly disagree	0	0	
*I know who is and is not eligible for no-cost contraception through FPE*			**0.02**
Strongly agree	27 (47.4%)	43 (75.4%)	
Agree	22 (38.6%)	11 (19.3%)	
Neutral	6 (10.5%)	3 (5.3%)	
Disagree	2 (3.5%)	0	
Strongly disagree	0	0	
*My clinic can easily provide all methods of reversible contraception to everyone who wants them*			**0.05**
Strongly agree	33 (57.9%)	45 (79.0%)	
Agree	18 (31.6%)	11 (19.3%)	
Neutral	4 (7.0%)	0	
Disagree	2 (3.5%)	1 (1.8%)	
Strongly disagree	0	0	
*I am familiar with the challenges that clients can experience when seeking contraception services*			0.13
Strongly agree	31 (54.4%)	42 (73.7%)	
Agree	24 (42.1%)	15 (26.3%)	
Neutral	1 (1.8%)	0	
Disagree	1 (1.8%)	0	
Strongly disagree	0	0	
*I can help a client get any contraceptive method they want*			**0.03**
Strongly agree	32 (58.2%)	45 (80.4%)	
Agree	10 (18.2%)	8 (14.3%)	
Neutral	11 (20%)	2 (3.6%)	
Disagree	2 (3.6%)	1 (1.8%)	
Strongly disagree	0	0	
*I would help a client get any contraceptive method they want, even if I think they should be using something else*			0.12
Strongly agree	41 (71.9%)	50 (87.7%)	
Agree	8 (14%)	4 (7%)	
Neutral	7 (12.3%)	3 (5.3%)	
Disagree	1 (1.8%)	3 (5.3%)	
Strongly disagree	0	0	
*Contraceptive services are an important part of healthcare*			0.40
Strongly agree	53 (93%)	55 (96.5%)	
Agree	4 (7.0%)	2 (3.5%)	
Neutral	0	0	
Disagree	0	0	
Strongly disagree	0	0	
*Contraceptive services are an important part of my job*			0.24
Strongly agree	48 (84.2%)	52 (91.2%)	
Agree	4 (7%)	4 (7%)	
Neutral	5 (8.8%)	1 (1.8%)	
Disagree	0	0	
Strongly disagree	0	0	

### Adoption

Post-simulation evaluations were positive, with most participants (84.1%) reporting that the simulation exceeded their expectations; that they would be able to apply what they learned in their clinical work (84.4%); and that they would strongly recommend the simulation training to a colleague (81.3%).

### Implementation

The content of the simulations remained consistent across implementation; however, several aspects of the simulations changed in response to various circumstances. As mentioned, the COVID-19 pandemic limited the number of clinic staff participating in each simulation training to six. For two sites, we limited the simulation to the first scenario to accommodate time limitations. For clinics with an onsite pharmacy (*n* = 2), the end of the second simulation differed from sites without an onsite pharmacy and allowed for pharmacist participation and simulated dispensing of emergency contraception. Finally, for the final participating site (Southeastern clinic), we implemented simulation training at the outset of their partnership with FPE, rather than at the one-year marker. We did this due to feedback received from other participating sites that they wished they’d had access to simulation training earlier in the program, rather than at the program midpoint.

### Maintenance

Assessments of monitoring data before and after the simulation training did not show increases in either the actual number of FPE clients nor the proportion of FPE clients to total contraceptive clients. At the end of the training, all clinic participants and FPE programmatic representatives were ask to reflect on the learnings of the day, and generated a list of FPE program- and clinic-specific goals through a facilitated action planning session. A summary of the items identified during the simulation action planning and their resulting programmatic changes is provided in Table 4.

**Table 4:** Family Planning Elevated programmatic changes made as a result of simulation training action planning

Clinic Identified Needs/Actions	FPE Programmatic Responses triggered by action planning session
Additional resources for educating patients on the full range of contraceptive methods	Provided each clinical site with birth control method demo kits
	Created a contraceptive methods patient information sheet
Additional information about how to implement FPE	Distributed and educated on the program eligibility job aid
	Supported clinics who were new to contraceptive billing and coding with job aids and technical assistance calls
	Created a “graduation plan” for exiting clinics to reference regarding end-of-program expectations and resources
	Provided exit survey cards and posters for clinics needing additional support in administering FPE’s client exit survey,
Opportunities for providers and support staff to improve their clinical skills	Introduced a new simulation training on the provision of emergency contraception
	Provided follow-up IUD training, in-person and virtual training on barrier methods, hosted two live webinars on barrier and knowledge-based methods, and connected teams with monthly training opportunities from partner organizations
Expansion of the contraceptive methods available through their clinic (and FPE)	Shared information about new-to-market methods and current payer coverage
	Worked with local hospital systems to explore coverage of postpartum and interval tubal ligation at low cost
Explore/pilot Dispensing Medical Practitioner licensing for in-clinic dispensing of prescription contraception	Provided technical guidance to three clinics interested in pursuing a license to dispense prescription-based hormonal contraception
Report on FPE’s research findings and information on how that data will be used to change state-level reproductive health policy	Created customized data reports for each clinic, providing a summary of the clinic’s service delivery data during their FPE enrollment
	Posted bi-annual policy briefs on the organization website and shared these links in a quarterly newsletter
Additional support on advertising the FPE program to clients in English and Spanish	Collaborated with clinics to develop and implement a tailored media campaign (online, print, radio, etc.)
	Developed cards describing the program for clinics to display in their waiting rooms, and for staff to share at the front desk
	Increased marketing in Spanish

## Discussion

Implementing a novel program with heterogeneous partners can be challenging. Results of this pilot study suggest that highly realistic in-situ simulation can be used as a means of assessing implementation fidelity. Additionally, simulation is useful not only in identifying gaps in program implementation, but also in working with clinics to understand *why* the program was not implemented correctly, and how to address those issues. Specifically, simulation is potentially useful in both investigating systems-level gaps and understanding individual knowledge and skill gaps. Previous studies on the use of quality audits have found that they are best received when the feedback is provided by a supervisor or colleague, rather than an external individual or body [11]. Simulation provides a unique approach to feedback, as participants are encouraged to self-reflect with their colleagues on the simulated performance and engage in a collaborative in-person discussion with the program team. Future research comparing the acceptability of simulation training to traditional means of evaluating implementation fidelity (self-report, observations, quality audits) would be useful in understanding the full potential of simulation in this area.

One of the main theoretical strengths of a simulation approach is that it provides benefits to both the clinical and the program teams. The clinical team have opportunities to practice team communication, counseling and technical skills, and receive feedback from clinical experts. The programmatic team gains insight into structural, personnel, and programmatic challenges to implementation both by observation of the program in action and through discussion with the implementing team. These trainings were viewed positively by the clinical team and simulation participants reported improved self-efficacy and knowledge about the FPE program.

Additionally, we found simulation an important feedback mechanism to improve program design and implementation. For example, the simulations illuminated that: 1) universally, clinics struggled to determine client eligibility for the FPE CAP program; and 2) even when clinics did understand eligibility components of the program, there were clients who could significantly benefit from program participation, but who fell outside of the eligibility criteria. The simulations were a useful backdrop to provide clinic education and support for addressing eligibility, but also led to the FPE team making internal changes to the program to better meet clinic and client needs (which of course differed across clinical sites). The tension between programmatic fidelity and flexibility is not a new finding. A 2010 study on implementation fidelity found that perfect adherence to the intervention protocol was less predictive of good intervention outcomes than a moderate level of adherence, and pointed to the need for practitioner flexibility and interventions that adapt to the context of the setting [7]. The use of simulation and the debriefing sessions allowed us to both observe and discuss where program adaptation could lead to better program outcomes.

Our study did not find changes in the number or proportion of FPE clients served at clinical sites following the simulation training. There are several potential reasons for this. First, it is possible that the simulation training penetrance was not sufficient to make significant changes in program implementation. It is unknown what exact threshold of training (number of clinical personnel trained in a single setting) is needed to ensure there is diffusion of the concepts enough to create the cultural change needed to sustain clinical practice changes. As programmatic assessment, not clinical behavior change, was the main purpose of these simulation trainings, we did not conduct a study design with multiple simulations to assess whether the number of exposures to the simulation training was associated with changes in program implementation, but future research should assess this dose/response relationship. It is also possible that the clinical adaptations in response to the ongoing COVID-19 pandemic limited our ability to determine change pre- and post- simulation exposure. As previously noted, simulation trainings largely took place after March 2020 and many sites explained that they changed practices in response to pandemic limitations, which ultimately affected the number of clients they could see. Finally, and importantly, the simulations were conducted in the middle of the intervention. Much of the learnings that lead to program adaptations focused around the creation of additional supporting materials for the clinics and “exception” clarifications that could have resulted in increased utilization had they been identified and adapted earlier on in the program implementation. Ultimately, we learned that simulation earlier, with increased dosing and for a wide audience may have the potential to influence program ultization.

As with all studies, this study has several limitations. First, while clinics were obligated to participate in simulations as part of their program requirements, the providers who attended the simulations chose to do so themselves or were selected by program administrators. It is thus possible that selection bias influenced those who felt favorably about the FPE program and its mission to participate in the trainings. Second, group size limitations, due both to the pandemic and simulation in general, meant that the entire clinic team did not participate in simulation trainings, which limited the generalizability on the reach of this type of intervention within clinical sites. Additionally, although timing and logistical constraints meant that some sites were limited to only the first scenario, we did not have a large enough sample size to stratify results in a meaningful way by the number of simulations. As a result, there may be a dose/response relationship between the amount of simulation and its impact that we are unable to determine. Further research is needed to understand the role simulation can play in the introduction of new clinical protocols and to understand the dissemination of that innovation across a clinical team and a clinic system.

## Conclusions

Highly realistic family planning simulations that occur in the clinical setting are acceptable to participants, positively change knowledge and clinical team confidence, and can identify systems gaps in clinical care and program implementation. Working with low-cost materials, facilitated simulation, debriefing and action planning can be used to assess implementation fidelity in a way that is acceptable, non-threatening and effective. This could be relevant to many programs that are looking for ways to monitor program implementation fidelity in ways that are reciprocal and beneficial to the implementation teams as well as providing an opportunity for feedback and collaborative iterative growth of a program to meet the needs of the implementers.

## Supplementary Information

**Additional file 1: **

**Additional file 2: **

## Data Availability

The datasets used and analyzed during this study are available for from the corresponding author (susanna.cohen@hsc.utah.edu) on reasonable request.

## Abreviations

CFIR: Consolidated Framework for Implementation Research
EHR: Electronic Health Record
FPE: Family Planning Elevated
FPE CAP: The Family Planning Elevated Contraceptive Access Program
IF: Implementation Fidelity
IUD: Intrauterine Devise
MA: Medical Asisstant
SP: Simulated Patient
