# Supplementary material for: Facility-based simulation as a programmatic tool for implementing a statewide contraceptive initiative
Source: BMC Health Serv Res. 2022 Jul 29;22:965. doi: 10.1186/s12913-022-08332-4 (PMC9335456; doi:10.1186/s12913-022-08332-4)
Supplement: Supplementary file 1 — Additional file 1: [file 12913_2022_8332_MOESM1_ESM.pdf]

Simulation Global Scores

The purpose of a global score is to capture the rater's overall impression of how well or poorly the simulation participant met the description of the dimension being measured. The global scores reflect holistic evaluation of the simulated participant, which cannot necessarily be separated into individual elements.

Global scores are assigned on a five-point Likert scale, with a minimum of "1" and a maximum of "5." The coder assumes a default score of "3" and moves up or down as indicated. A "3" may also reflect mixed outcomes. A "5" is generally not given when there are any examples of poor practice in the simulation.

Global scores should be assigned immediately after viewing of the simulation and prior to tallying results of any checklist components.

Treats Client with Respect				
Low				High
1	2	3	4	5
Participant actively demeans or belittles client's knowledge, intelligence, or identity	Participant demonstrates disinterest in client, does not listen to what the client says, or interrupts client	Participant superficially listens and responds to client, but does so in a lukewarm or erratic fashion	Participant fosters collaboration and active listening to client. Demonstrates interest in client's needs.	Participant fosters collaboration, active listening, and deep interaction with client that affirms client's knowledge, intelligence, or identity.

Demonstrates Programmatic Adherence				
Low				High
1	2	3	4	5
Participant does not know about FPE, provides the opposite of programmatic guidance and increases barriers to program for client	Participant demonstrates limited adherence to programmatic guidelines and does not appear to understand the main tenants of the program	Participant understands and abides by the main tenants of the program, but cannot apply the program to specific scenarios	Participant understands and abides by the main tenants of the program and demonstrates active attempts to apply it to individual circumstances and scenarios	Participant understands and abides by all tenants of the program and demonstrates proficiency in applying the program to all circumstances and scenarios

Simulation Checklist(s)

***Prior to coding the simulation, any modifications implemented during the simulation (e.g., whether or not the simulated patient asks about something specific or whether the clinic has specific restrictions) should be noted so that they can be scored N/A on the checklist items.**

Front desk staff scenario 1:

	Yes	Partial	No	N/A	Notes
Greets/welcomes the client					
Identifies client's income as Medicaid eligible					
Understands the client is undocumented/not Medicaid eligible					NOTE: This could simply be how the staff responds to Gabi presenting her Mexican passport
Demonstrates professionalism around client's documentation status					NOTE: A "No" would be forcing someone to disclose aloud that they are undocumented. A "partial" might involve proxy questions, like "Were you born here?" or something similar.
Tells client they qualify for a program that covers the costs of their contraceptive visit and the contraceptive method of their choice (does not have to mention FPE)					NOTE: A "Partial" would be if someone says ONE thing (program exists and is covered; OR; that the method of their choice is covered, but not both.
Assures client that the program does not count as government assistance					NOTE: Asking if the program counts as government assistance is optional and may not occur.
Total					
Session Notes:					

****COVID may impact how clinics utilize their front-desk staff***

GLOBAL SCORES

Treats Client with Respect				
Low				High
1	2	3	4	5
Participant actively demeans or belittles client’s knowledge, intelligence, or identity	Participant demonstrates disinterest in client, does not listen to what the client says, or interrupts client	Participant superficially listens and responds to client, but does so in a lukewarm or erratic fashion	Participant fosters collaboration and active listening to client. Demonstrates interest in client’s needs.	Participant fosters collaboration, active listening, and deep interaction with client that affirms client’s knowledge, intelligence, or identity.

Demonstrates Programmatic Adherence				
Low				High
1	2	3	4	5
Participant does not know about FPE, provides the opposite of programmatic guidance and increases barriers to program for client	Participant demonstrates limited adherence to programmatic guidelines and does not appear to understand the main tenants of the program	Participant understands and abides by the main tenants of the program, but cannot apply the program to specific scenarios	Participant understands and abides by the main tenants of the program and demonstrates active attempts to apply it to individual circumstances and scenarios	Participant understands and abides by all tenants of the program and demonstrates proficiency in applying the program to all circumstances and scenarios

Medical Assistant or other Healthcare staff scenario 1:

	Yes	Partial	No	N/A	Notes
Introduces themselves to the client					NOTE "Yes" would equal introducing yourself by name; "Partial" would be a greeting, but no specific introduction;
Confirms with client that the FPE visit is free of charge (doesn't have to mention FPE by name)					May or may not occur
Reassures client that the program (FPE) does not count as government assistance					May or may not occur
Confirms client can receive same-day services/method					
Confirms client can select method of their choice					NOTE: A "Partial" might be if the MA provides information about methods, but doesn't explicitly confirm that FPE covers all methods (except tubal)
Provides patient with a decision aid presenting method options					NOTE: A "Partial" might be a terrible, old, outdated decision-aid. Please try to note the type of decision aid in the Notes section if you can see it (e.g., Bedsider, FPE method, new method). A decision aid could be a handout, a website, a demo of methods, a poster, etc.
Primes patient to think about contraceptive priorities					NOTE: A "Yes" would involve some reflective listening about what the patient has said is important to her about a contraceptive method. A "Partial" would involve just giving a methods sheet but not really delving into any attributes or features or reflective conversation about methods; A "No" would include just asking what method the patient wants without any further discussion.
Shares clinically **accurate** information					NOTE: A "Yes" would require that everything the MA says is clinically accurate. A "Partial" would be if some things are accurate and some are not. A "No" would mean that nothing the MA said was fully accurate. Misinformation includes over- or understating risks or side effects or generalizations about what most people do.
Provides **correct information** about FPE					
Total					
Session Notes:					

COVID may impact how clinics utilize their MAs

GLOBAL SCORES

Treats Client with Respect				
Low				High
1	2	3	4	5
Participant actively demeans or belittles client's knowledge, intelligence, or identity	Participant demonstrates disinterest in client, does not listen to what the client says, or interrupts client	Participant superficially listens and responds to client, but does so in a lukewarm or erratic fashion	Participant fosters collaboration and active listening to client. Demonstrates interest in client's needs.	Participant fosters collaboration, active listening, and deep interaction with client that affirms client's knowledge, intelligence, or identity.

Demonstrates Programmatic Adherence				
Low				High
1	2	3	4	5
Participant does not know about FPE, provides the opposite of programmatic guidance and increases barriers to program for client	Participant demonstrates limited adherence to programmatic guidelines and does not appear to understand the main tenants of the program	Participant understands and abides by the main tenants of the program, but cannot apply the program to specific scenarios	Participant understands and abides by the main tenants of the program and demonstrates active attempts to apply it to individual circumstances and scenarios	Participant understands and abides by all tenants of the program and demonstrates proficiency in applying the program to all circumstances and scenarios

Provider scenario 1:

	Yes	Partial	No	N/A	Notes
Introduces themselves to the client					NOTE "Yes" would equal introducing yourself by name; "Partial" would be a greeting, but no specific introduction;
Sits at client level, facing client with open body language					NOTE: A "Partial" might include provider exhibiting at least one of these things: signs of impatience (tapping foot, pen, etc), turning away a lot to face the computer, arms crossed. A "No" might include the provider exhibiting one or more of these things (or otherwise standing, pacing, etc.)
Uses open-ended questions, where appropriate					NOTE: A "Yes" would involve the provider mostly asking open-ended questions, particularly about patient preferences and values. A "Partial" would involve if the provider mostly asked close-ended questions and those close-ended questions were specifically around patient preferences. A "No" would involve not having any open-ended questions in the conversation.
Asks client about their reproductive goals					
Asks client about contraceptive preferences [hormones v. side effects, how often wants to interact with method, privacy, fertility return, etc.]					NOTE: A "Partial" would involve focusing on one aspect of preference, not linking preference to the method being discussed, and not discussing other methods that align with that preference.
Responds respectfully/non-judgmentally when client reports wanting to have a menstrual period					NOTE: A "Partial" score would involve making some judgmental comments or "educational attempts" but ultimately allowing that the patient's preference is important and not pushing the matter.
Asks questions about client's previous contraceptive experiences					NOTE: A "Partial" score would involve only going to the most recently-used method and discussing that. A "Yes" would be delving deeper into contraceptive experiences in life and tying it back to preferences.
Provides/Uses visual or kinesthetic options for learning about methods [e.g., handout or physical examples of methods]					NOTE: A "yes" would involve using the visual/kinesthetic aid as an active part of the discussion (referring to it, pointing to it, letting people touch method demos, etc). A "Partial" would involve any provision

					or reference to the method sheet, but not using it to visually show anything or further the discussion.
Is able to correctly answer all the client's questions about contraceptive methods in an affirming, normalizing manner appropriate to the patient's health literacy level					NOTE: A "Partial" would involve demonstrations of either bias toward a method(s) or overmedicalizing something whilst being affirming.
Helps the client weigh the pros and cons of the method(s) of interest					NOTE: A "No" would be only listing the pros of one method and only listing the cons of another. A "Partial" would include not being complete/realistic in discussion the pros and cons of a method, but rather focusing on one particular aspect and providing that.
Demonstrated reflective listening with the client					NOTE: A "Yes" would include statements that acknowledge what the client said, statements that remember what the client preferences are, etc.
Clearly explained how to USE the method chosen by the client					NOTE: "Yes" for the IUD would include information about insertion and removal as well as other components such as length of use, trimming the strings, partner feeling strings, and what to expect immediately after insertion etc. A "Partial" would involve some discussion of either insertion or removal but not both, leaving out information about what to expect in the first six months.
Told the client about the health risks of the method chosen					NOTE: A "Yes" for an IUD would need to involve discussion about perforation, expulsion, PID, potential ectopic pregnancy. A "Partial" would involve discussion about one thing.
Told client about potential side effects of the method chosen					NOTE: A "Yes" for an IUD would be related to other side effects beyond bleeding (yeast infections, UTIs, pain during sex, pain during exercise, cramping, breast tenderness, bacterial vaginosis). A "Partial" for this would be focused solely on bleeding expectations.
Confirms that all methods, follow-up visits, and removal services are free of charge through FPE					NOTE: A "Yes" would involve the provider affirming that a person could both switch or discontinue at no charge throughout the program. A "Partial" would be just noting that removal would be covered.
Confirms that FPE is not government assistance [responds to client's documentation status concerns]					

Willing to book same-day appointment for insertion					NOTE: A “Partial” would be where the provider indicates that same-day services are possible, but isn’t sure whether they are possible or the client at that time. A “Yes” would be affirming that they do same day and that the client can get same day.
Discussed with the client what to do if the client wanted to change or stop using the chosen method					NOTE: A “Yes” would require that the provider affirmed that the client could get the IUD removed for any reason (including not liking it).
Shared clinically **accurate** information					NOTE: A “Yes” would require that everything the provider says is clinically accurate. A “Partial” would be if some things are accurate and some are not. A “No” would mean that nothing the provider said was fully accurate. Misinformation includes over- or understating risks or side effects or generalizations about what most people do.
Shared **correct information** about FPE					
Which methods did the provider discuss with the client? (check all that apply)	○ Implant<br>○ Hormonal IUD<br>○ Copper IUD<br>○ Pills<br>○ Patch<br>○ Ring<br>○ Vasectomy<br>○ Tubal ligation		○ Depo<br>○ Condoms<br>○ Diaphragm<br>○ Cervical cap<br>○ Sponge<br>○ FABM<br>○ Withdrawal<br>○ Spermicide<br>○ Ella<br>○ LNG EC<br>○ Copper IUD as EC		
Total					
Additional provider session notes:					

GLOBAL SCORES

Treats Client with Respect				
Low				High
1	2	3	4	5
Participant actively demeans or belittles client’s knowledge, intelligence, or identity	Participant demonstrates disinterest in client, does not listen to what the client says, or interrupts client	Participant superficially listens and responds to client, but does so in a lukewarm or erratic fashion	Participant fosters collaboration and active listening to client. Demonstrates interest in client’s needs.	Participant fosters collaboration, active listening, and deep interaction with client that affirms client’s knowledge, intelligence, or identity.

Demonstrates Programmatic Adherence				
Low				High
1	2	3	4	5
Participant does not know about FPE, provides the opposite of programmatic guidance and increases barriers to program for client	Participant demonstrates limited adherence to programmatic guidelines and does not appear to understand the main tenants of the program	Participant understands and abides by the main tenants of the program, but cannot apply the program to specific scenarios	Participant understands and abides by the main tenants of the program and demonstrates active attempts to apply it to individual circumstances and scenarios	Participant understands and abides by all tenants of the program and demonstrates proficiency in applying the program to all circumstances and scenarios

Debrief Codebook and Instructions

<u>Purpose of a simulation debrief:</u>
A simulation debrief provides two essential functions: a) it allows participants to reflect on their actions/behaviors during the simulation, assess them, and identify if there are areas for change; and b) it provides the program team insight into underlying beliefs, structural and environmental challenges, and procedural barriers that affect programmatic implementation. The purpose of this codebook is only to assess the success of the second function, as participant experience and assessment is best captured through individual feedback. By coding the content provided in the debrief, the true programmatic usefulness of the debrief can be aggregated and further understood.

<u>Instructions for coding a simulation debrief:</u>

Debrief conversations should be coded via utterances. An utterance is defined as a complete thought or a thought unit. Each utterance should receive only one code. For example, "*Our clinic doesn't accept FarmHealth insurance"* is an utterance that would be assigned one code, whereas, "*Our clinic doesn't accept FarmHealth insurance so I don't know how I would help her get a no-cost IUD*" could be parsed into two utterances that would each receive a separate code.

In coding the simulation debrief, only code utterances provided by the participant group, not the simulation/programmatic staff.

It is suggested that coders code the timestamps of utterances to help with later coding comparisons between coders.

Utterances should be coded according to the following categories. An utterance can be positively (+) or negatively (-) valenced, depending on the context. When inserting a code, ensure that you code both for the utterance AND for the valence (+/-).

**Programmatic Category and code**	**Definition**	**Examples/Notes**
Broad Education/awareness **(BEA)(+/-)**	Statement/reflection that identifies education or awareness (or lack thereof) about broader issues related to family planning, but NOT specific to the FPE program or clinical knowledge	E.g., "I didn't realize Medicaid accepts people up to 138% federal poverty level."
FPE programmatic knowledge/awareness **(PKA)(+/-)**	Statement/reflection that denotes programmatic education/awareness (or lack thereof) specifically about the FPE program	E.g., "I didn't know that you guys allowed us to offer the program to teens"
Bias **(B)(+/-)**	Statement/reflection that denotes participant bias toward patients, methods, timing/context of contraceptive provision, or strong personal preferences for what the participant believes should be done in a particular clinical scenario.	E.g., "I mean, I'd feel very reluctant if someone asked for their IUD out but didn't want to get on something else."
External systems barriers **(ESB)(+/-)**	Statement or reflection that identifies external systems/policy or legal barriers to program implementation	E.g., "Since the law requires parental consent to give methods to teens, we've opted to not use the FPE program among adolescents."
Internal systems barriers **(ISB)(+/-)**	Statement or reflection that identifies internal systems barriers to program implementation (e.g., clinic workflow, scheduling, stocking, communication)	E.g., "I think the problem is that we have such high staff turnover, few people remember that the tablets are there and that they should be offering the surveys to all clients."
Clinical knowledge gaps **(CKG)(+/-)**	Statements/reflections that identify gaps in clinical knowledge/education	E.g., "Well, my understanding is that the morning after pill causes the termination of a pregnancy."
