# Supplementary material for: Facility-based simulation as a programmatic tool for implementing a statewide contraceptive initiative
Source: BMC Health Serv Res. 2022 Jul 29;22:965. doi: 10.1186/s12913-022-08332-4 (PMC9335456; doi:10.1186/s12913-022-08332-4)
Supplement: Supplementary file 2 — Additional file 2: [file 12913_2022_8332_MOESM2_ESM.pdf]

SIMULATION POST-SURVEY

Thank you for your involvement in the Family Planning Elevated Contraceptive Assessment Program. Your employer has asked that you participate in these simulation training sessions which are being conducted to identify strengths and weaknesses in the contraception counseling programs at each of the FPE CAP sites.

We would like to ask you to complete a brief survey about your experience with the simulation training sessions. We have a survey that we will ask you to complete before the simulation training and then one after you have completed the simulation training. The surveys will take you approximately 5 minutes each and you will be able to submit these anonymously.

There are no known risks to your participation in these surveys and there is no direct benefit to you. However, the information you provide will assist is un planning our training sessions for the FPE Program.

If you have any questions complaints or if you feel you have been harmed by this research please contact Rebecca Simmons, PhD at the University of Utah at (801)-581-6170.

Contact the Institutional Review Board (IRB) if you have questions regarding your rights as a research participant. Also, contact the IRB if you have questions, complaints or concerns which you do not feel you can discuss with the investigator. The University of Utah IRB may be reached by phone at (801)-581-3655 or by e-mail at irb@hsc.utah.edu.

Thank you for your willingness to help!

First, we would like be able to anonymously assess your responses before and after you participate in the simulation. To do so, we'll ask you to create an identification code that can be used to match your pre-simulation survey with your post-simulation survey. To do this, please put the following letters and numbers in the five boxes below:

1. The number of sisters you have (e.g., 0)
2. The last letter of your mother's first name (e.g., 'Mary' would be 'Y')
3. The fifth digit of your phone number (e.g., '801-566-3089' would be '6')
4-5. The month digits of the year you were born (e.g., 'April' would be '04')

A person's code using our examples would be **0Y604**

1. Where did you attend the simulation training?

2. Have you ever participated in simulation training before?
   - ○ Yes (go to question 3)
   - ○ No (go to question 4)
   - ○ Not sure (go to question 4)

© 2020

3. How was your previous experience with simulation training?
- o Excellent
- o Good
- o Fine
- o Bad
- o Terrible

4. What are you expecting from today's simulation training? [Anything you expect to learn? Anything you are worried about? Anything you are excited about?]

5. Please rate your level of agreement with each of the following statements:

	**Strongly Agree**	**Agree**	**Neutral**	**Disagree**	**Strongly Disagree**
I have a good understanding of how contraceptive care is provided at my clinic (including how it occurs when I am not around)					
I have a good understanding of how the Family Planning Elevated program works within my clinic					
I know who is and is not eligible for no-cost contraception through the Family Planning Elevated program					
My clinic can easily provide all methods of reversible contraception to everyone who wants them					
I am familiar with the challenges that clients can experience when seeking contraception services					
I can help a client get any contraceptive method they want					
I would help a client get any contraceptive method they want, even if I think they should use something else					
Contraceptive services are an important part of healthcare					
Contraceptive services are an important part of my job					

 © 2020

Thank you for participating in the simulation today and for providing us with valuable feedback about your experiences. To match your survey with your pre-survey, please complete the 5-digit code again, so we can match your responses. To do this, please put the following letters and numbers in the five boxes below:

1. The number of sisters you have (e.g., 0)
2. The last letter of your mother's first name (e.g., 'Mary' would be 'Y')
3. The fifth digit of your phone number (e.g., '801-566-3089' would be '6')
4/5. The month digits of the year you were born (e.g., 'April' would be '04')

A person's code using our examples would be **0Y604**

1. Please rate your level of agreement with each of the following statements:

	Strongly Agree	Agree	Neutral	Disagree	Strongly Disagree
I have a good understanding of how contraceptive care is provided at my clinic (including how it occurs when I am not around)					
I have a good understanding of how the Family Planning Elevated program works within my clinic					
I know who is and is not eligible for no-cost contraception through the Family Planning Elevated program					
My clinic can easily provide all methods of reversible contraception to everyone who wants them					
I am familiar with the challenges that clients can experience when seeking contraception services					
I can help a client get any contraceptive method they want					
I would help a client get any contraceptive method they want, even if I think they should use something else					
Contraceptive services are an important part of healthcare					
Contraceptive services are an important part of my job					

 © 2020

2. Please rate your experiences with the simulation training today:

	**Strongly Agree**	**Agree**	**Neutral**	**Disagree**	**Strongly Disagree**
The training felt applicable to the experiences and interactions I have as part of my job					
I will be able to apply something I learned in today's simulation to my future interactions with family planning clients					
I have a better understanding of what makes "patient-centered" care after today's simulation					
I am confident that I can provide family planning clients with a more "patient-centered" experience as a result of today's training					
I would recommend this simulation experience to my colleagues if they have not yet taken it					

3. How did the simulation experience match your expectations?
- ○ Exceeded them (better than I expected)
- ○ Met them (about what I expected)
- ○ Fell short of them (worse than I expected)
- ○ Was a complete waste of time (I hated it!)
- ○ Other (please explain)____

4. What was your main takeaway from this simulation experience?

Thank you for your participation!

 © 2020
